# Gait Analysis before and after Gastrocnemius Fascia Lengthening for Spastic Equinus Foot Deformity in a 10-Year-Old Diplegic Child

**DOI:** 10.1155/2010/417806

**Published:** 2010-03-22

**Authors:** Manuela Galli, Veronica Cimolin, Giorgio Cesare Santambrogio, Marcello Crivellini, Giorgio Albertini

**Affiliations:** ^1^Bioengineering Department, Politecnico Di Milano, Piazza Leonardo da Vinci 32, 20133 Milano, Italy; ^2^IRCCS “San Raffaele Pisana”, Tosinvest Sanità, Via della Pisana 235, Roma, Italy

## Abstract

*Purpose*. This case study quantified kinematic and kinetic effects of gastrocnemius lengthening on gait in a Cerebral Palsy child with equinus foot. *Methods*. A 10-year-old diplegic child with Cerebral Palsy was evaluated with Gait Analysis (GA) before and after gastrocnemius fascia lengthening, investigating the lower limb joints kinematics and kinetics. *Results*. Kinematics improved at the level of distal joints, which are directly associated to gastrocnemius, and also at the proximal joint (like hip); improvements were found in ankle kinetics, too. *Conclusions*. This case study highlighted that GA was effective not only to quantify the results of the treatment but also to help preoperative decision making in dealing with CP child.

## 1. Introduction

One of the most common deformities in children with spastic Cerebral Palsy (CP) is equinus foot, characterised by a reduced dorsiflexion control during gait. It is the result of muscle imbalance between overactive plantarflexors (gastrocsoleus) and normal or weakened dorsiflexors. Equinus is often dynamic with no fixed contractures. As the child develops a mature gait, fixed equinus contracture can develop, predominately of the gastrocnemius [1–4].

Recent studies demonstrated that in patients with this limitation, the muscles of the ankle participating in posture and locomotion present an impairment in controlling ankle torque during postural adjustment in quit stance and propulsion during terminal stance and clearance during the swing phase. In particular it was demonstrated that in CP patients, dorsiflexion torque steadiness, which refers to the ability to perform voluntary muscle contractions with minimum fluctuations in torque while matching a given torque level, is related to agonist and antagonist muscle activation variability as well as the amplitude of antagonist coactivation, unlike healthy children where it is related only with agonist muscle activation variability. In these patients ankle function is impaired during isometric contractions, as they exhibited reductions in torque steadiness and maximal voluntary torque of both dorsi- and plantarflexors in comparison to healthy children [5]. 

This persistent deformity interfering with function is treated with the surgical lengthening of the triceps surae, in particular in children aged between 6 and 12 [6]. Muscle-tendon lengthening operation, at an early age, is known to increase the risk of recurrence and the need for repeated surgery [7]. 

Considerable debate has occurred about surgical correction of equinus gait in children with CP. A large number of procedures have been described for operations on equinus foot. In particular two different kinds of surgical interventions are generally performed in order to restore normal ankle motion and function: one type of operation alters the length of both the gastrocnemius and soleus muscle-tendon unit [3, 8–13]; another one modifies the length of the gastrocnemius muscle-tendon unit without altering soleus [14–18]. It is still unknown and controversial which technique is the more appropriate for each patient. Some clinicians routinely lengthen only the gastrocnemius and not Achilles tendon, in order to avoid the risk of excessive ankle dorsiflexion and iatrogenic crouch in some patients. However, rates of recurrence and calcaneous are noticed in both procedures. Unfortunately the debate about overlengthening/weakening is based on extremely limited observational data [19–21]. In addition, although both procedures typically improve ankle kinematics over the gait cycle [13–23], they both may also weaken the plantarflexor muscle, decreasing ankle power during gait stance phase [24]. 

In literature the gait changes after triceps surae lengthening for spastic equinus foot reduction have been mainly evaluated using 3D Gait Analysis (GA), a technique particularly suitable to value the results and effectiveness of the surgical treatment of equinus foot [1, 13, 18, 19, 23, 25–27]. But this type of operation has been generally performed during multilevel surgery, thence it was difficult to evaluate the effects of a single technique objectively; then, when the treatment was performed during single-level surgery, the attention was focused mainly on the joint related to the treated muscle (i.e., ankle joint if the treated muscle is the triceps surae) while other joints, not directly related to the treated muscle, were neglected (like hip joint) or poorly investigated (like knee joint). Moreover, all previous studies have mainly presented the quantitative outcomes obtained with GA, without focusing on the role of GA during the decision making process in the evaluation of patients' conditions.

In the present case study we also use GA to quantify the effects of the modified Vulpius gastrocnemius lengthening technique for the reduction of equinus foot deformity due to calf spasticity, but, at the same time, we wanted to point out how much it could support clinical decisions in the treatment of the CP child. His gait was assessed considering distal and proximal joints and kinematic and kinetic data. He was examined before the treatment, 2 months and 10 months after bilateral surgery. In this way it is possible to quantify the patient's gait strategy thoroughly.

## 2. Case Presentation

The analysed subject was a 10-year-old male affected by diplegic Cerebral Palsy; he was born approximately 7 weeks preterm with respiratory distress. When he came to our centre to be examined he showed a fixed contracture of the gastrocnemius and the soleus, as demonstrated by a negative Silfverskiold test (lack of dorsiflexion of the foot to neutral position with the knee in flexion and in extension) under anaesthesia. In addition, he presented a moderate contracture of hamstrings. He had undergone no previous surgery or other significant treatments for spasticity; he was an independent community ambulatory, using no assistive devices or orthoses. 

### 2.1. Patient Assessment

He was examined with a clinical multidisciplinary evaluation method and with 3D GA before (PRE session), 2 months (POST1 session) and 10 months (POST2) after bilateral gastrocnemius fascia lengthening surgery (modified Vulpius technique). This technique consists of a chevronlike incision of the gastrocnemius aponeurosis, followed by ankle dorsiflexion to neutral position and not beyond to avoid overlengthening [1].

The 3D GA was carried out using a 12-camera optoelectronic system (ELITE2002, BTS S.p.A., Milan, Italy) with passive markers positioned according to Davis et al. [28], for kinematic movement evaluation [29], two force platforms (Kistler, CH), for movement kinetic calculation, and a video system (BTS S.p.A., Milan, Italy) synchronic with the optoelectronic and force platform systems. After collecting some anthropometric measures (height, weight, tibial length, distance between femoral condyles or diameter of the knee, distance between malleoli or diameter of the ankle, distance between anterior iliac spines, and thickness of the pelvis), passive markers were placed at special reference points, directly on the subject's skin, as described by Davis et al. [28]. After the preparation phase, the patient was made to walk barefoot at his self-selected and convenient speed along a 10-metre walkway. In order to ensure consistency in the assessment of the trials, at least seven ones were recorded during each session. Starting from the 3D coordinates of the 3 markers placed on each segment (pelvis, thigh, leg and foot) a rigid frame was computed; the Euler angles between two frames are the flex-extension, ab-adduction and intra-extrarotation of principal joints (hip, knee, and ankle joints) [28].

Some indices obtained from kinematic and kinetic data were analysed: spatio-temporal parameters, joint-angle values at a specific instant of the gait cycle, and moment and power graphs (Table 1). The mean values (standard deviation) of kinematic and kinetic parameters are detailed, respectively, in Tables 2 and 3, in PRE and POST sessions and for normal range (CG). 

A control group of 15 healthy subjects (Control Group: CG; range: 7–12 years) was included. Selection criteria for nondisabled subjects included no prior history of cardiovascular, neurological, or musculoskeletal disorders. They exhibited normal ROM and muscle strength and had no apparent gait abnormalities.

### 2.2. Postoperative Management

A standard regime was used for rehabilitation after operation. Passive movement practise was started after five days and gait was trained for eight days. Immobilisation in plaster cast lasted five weeks and was followed by physical therapy, consisting of passive and active joint movements. In particular, the child was treated with an individual rehabilitation program, which initially included three sessions of physical therapy per week, starting six weeks after surgery and continuing for twelve weeks. The child gradually was advanced from a passive to an assisted range-of-motion protocol and finally to a resistance program. The frequency of physical therapy sessions was reduced to one per week after six months.

### 2.3. Statistical Analysis

A one-way between-groups analysis of variance (ANOVA) was applied to compare PRE, POST1, and POST2 sessions; the assumptions of the ANOVA model were tested by evaluating the fit of the observed data to normal distribution (Kolmogorov-Smirnov test) and homogeneity of variances (Levene's test). Specific effects were evaluated by means of the post-hoc comparisons of means (Bonferroni test). Null hypotheses were rejected when probabilities were below 0.05.

**Pre Session**

The child walked with a slightly low velocity of progression (0.6 + 0.1 1/sec; CG: 0.9 + 0.2 1/sec), reduced anterior step length (right: 0.15 + 0.09 m and left: 0.22 + 0.12 m; CG: 0.42 + 0.16 m) and larger step width (154.0 + 15.4 mm; CG: 97.4 + 23.6 mm), if compared to normal data; this condition was likely due to the search for better stability and equilibrium during gait. No abnormalities were found in terms of stance phase duration (right: 56.9 + 1.9% and left: 57.5 + 2.5%; CG: 56.6 + 2.1%).

The patient presented excessive ankle plantarflexion during the whole gait cycle and low joint excursion bilaterally. Foot progression was quite normal during the gait cycle.

The knee was characterised by excessive flexion during stance (worse on the right side than the left one) and diminished maximum swing-phase knee flexion, which caused a decrease of range of motion bilaterally.

The hip flex-extension graph revealed excessive right hip flexion during the whole gait cycle, while the left side showed a normal angle at initial contact and high flexion during midstance; a restricted range of motion was evident bilaterally, because of the limited extension in midstance exhibited by both sides. The hip displayed slightly limited joint excursion compared to CG on the frontal plane; it was bilaterally intrarotated on the transversal plane during the whole gait cycle.

Pelvic tilt was characterised by a slightly posterior position with a higher pelvis excursion compared to control group. No significant anomalies were found on the frontal plane, while slightly low pelvis excursion was present on transversal plane.

Sagittal plane kinetic evaluation showed lack of the first rocker (Table 4) and a nonphysiological and early maximum of ankle moment; in particular the value of maximum ankle moment (Max ADPM index) was close to average data bilaterally, but it appeared in the first stance phase not during terminal stance (right: 10.5 + 2.6% and left: 10.5 + 0.7%; CG: 45.5 + 0.5%). Moreover, the lack of power generation at the push-off for the ankle occurred bilaterally. The knee joint was characterised by high flexor moment in stance bilaterally, with abnormal values of knee power absorption, mainly on the right side, if compared to average range. This strategy was due to high knee flexion during the whole gait cycle, more accentuated on the right side. The hip joint displayed higher flexor moment and higher generated power at initial stance on the right side in comparison with normality, because of the exaggerated right hip flexion showed by kinematics.

**POST1 Session [2 months after surgery]**

Velocity of progression decreased significantly (0.3 ± 0.1 1/s), while the other spatio-temporal parameters turned to be unchanged.

Ankle joints improved significantly and bilaterally, passing from the previous plantarflexed position to dorsiflexion; still the range of motion remained scanty. No significant changes occurred in terms of foot progression.

Bilateral knee joint flexion decreased significantly, showing a more physiological position during the stance phase, even if asymmetry between right and left sides was still evident; no significant changes occurred in the magnitude of maximum swing-phase knee flexion.

The hip decreased its flexion on the right side in particular at initial contact, exhibiting angle values in the average range; the left limb did not change its physiological position at initial contact but it worsened its flexion during midstance. No changes were displayed in the frontal and transversal planes.

After surgery pelvic tilt was in a more physiological position. The pelvis remained unchanged on the other planes of movement.

Kinetic data showed that timing of maximum at ankle moment had improved significantly (right: 46.1 + 3.6% and left: 45.5 + 2.9%) even if its magnitude had decreased bilaterally.

The maximum of ankle power increased slightly, remaining still lower than control group. The knee joint reduced its moment and power maximum values bilaterally, approaching the values of control group. Significant improvements were found at right hip kinetics, which reached values close to normal data.

**POST2 Session [10 months after surgery]**

Spatio-temporal parameters revealed an improvement in progression velocity (0.7 ± 0.1 1/s) if compared to POST1 session, which meant that a safer gait might be due to increased strength.

The ankle joint maintained the dorsiflexed position in stance phase and exhibited a significant improvement as concerns the range of motion bilaterally, even if still below healthy values.

The knee joint increased its flexion at initial contact and during loading response, exceeding normality; in swing phase the maximum of knee flexion improved significantly, displaying values close to normal data bilaterally. The appearance of crouch gait during stance phase in this session may be connected to the increasing hamstring contracture, which generally represents the major factor influencing recurrence of equinus, and to the weakening of the soleus.

At the proximal joints (such as pelvis and hip joints) no significant changes occurred. No other meaningful changes were recorded in terms of the other kinematic graphs.

Kinetic ankle parameters did not modify their values, remaining lower than normal data. Knee kinetics worsened, reaching values close to PRE session; this result was directly connected to the knee flexion increase, highlighted by kinematics. No significant changes occurred on the level of hip kinetics.

## 3. Discussion

In this study the kinematic and kinetic aspects of gait in a 10-year-old child with CP were evaluated using GA with a twofold aim: firstly, to illustrate an example of the key role of GA in the decision making process of a treatment in CP and secondly, to quantify the effects of gastrocnemius fascia lengthening during single-level surgery for the correction of equinus foot. In particular the analysis was carried out considering distal and proximal joints and in terms of kinematic and kinetic data, too. 

The examination of PRE session data pointed out anomalous spatio-temporal parameters and nonphysiological kinematics of ankle and knee joints, that may be connected to a calf muscle contracture. In addition, a recent study [5] demonstrated that in CP children dorsiflexion torque steadiness is related to both agonist and antagonist muscle activation variabilities as well as the amplitude of antagonist coactivation. Then, ankle function is impaired during isometric contractions as they exhibited reductions in torque steadiness and maximal voluntary torque of both dorsi and plantarflexion and this may explain the kinematic anomalies displayed by this patient. In this child we can observe an excessive hip flexion, too, due to the incorrect biomechanical position of the distal joints, which had to maintain stability and equilibrium during gait. Ankle kinetics indicated an early maximum with good magnitude of moment and lack of propulsion ability during terminal stance bilaterally. 

In the light of this evaluation, the surgeon's main concern was to decide which operation to perform on the calf muscle, in order to reduce the equinus foot. In fact, gastrocnemius lengthening might bring about further muscle weakening. The evaluation of push-off ability is generally fundamental in order to understand whether and when a surgical intervention is permitted. The patient evaluated in this case study revealed strong equinus foot deformity and lack of ankle power generation during terminal stance. For this reason an operation did not seem suitable: it might weaken the spastic muscle excessively, causing the patient's walking inability. On the other side the natural history of gait in patients with CP shows the deterioration of their walking ability with time and increasing pain in lower limb joints [30]; walking abnormalities, if not corrected, may also cause some additional vaulting patterns.

However, a deep evaluation of gait analysis graphs and, in particular, of ankle kinetics led clinicians to choose surgery. 

It is important to make some biomechanical remarks. A moment is defined in fact as a force acting at a distance around an axis of rotation, which produces an angular acceleration around that axis; these moments are made up of two components: a force and a lever arm. Therefore moments are equal to the magnitude of the force times the perpendicular distance from the rotation axis (length of the lever arm). According to this definition, the magnitude of maximum of ankle moment close to normality and the presence of equinus foot deformity, which produces a limited length of lever arm, are indicative of a satisfactory force of the triceps surae. 

At the same time, as power is obtained by multiplying the moment times the joint angular velocity, it is clear that the lack of ankle power generation is not connected to muscle weakness but to the limited angular velocity, calculated from the ankle angle on the sagittal plane [4].

Taking these biomechanical elements and GA data into consideration, bilateral gastrocnemius fascia lengthening surgery was performed with satisfactory results not only in terms of kinematics but also of kinetics. Firstly, two months after surgery (POST1) ankle and knee joints showed an improvement in the angles, even if their ranges of motion remained scanty. In the second follow-up (POST2) knee and ankle range of motion improved. The treatment and the following physiotherapy program reduced the dominance of ankle plantarflexors over antagonist muscles and tibialis anterior was not contrasted by the abnormal gastrocnemius longer activity. It is important to highlight the recovery of high knee flexion during stance phase in the second follow-up (ten months). This implies that it needs performing an operation on the knee, like hamstring lengthening, in order to correct knee position during gait. In fact, as previous studies have widely highlighted, increasing hamstrings contracture, which mainly influences recurrence of equinus and soleus weakening, may contribute to the occurrence of crouch gait [18, 21].

The outcomes of our case study showed that both the ankle joint and partially the knee (increment in ROM and knee flexion during swing phase) had improved as well as the hip, mainly on the right side.

The new biomechanical position of the ankle joint had direct consequences on ankle kinetics. Firstly, we noticed timing normalization of the maximum ankle moment (Max ADPM index) in POST1 session, despite a decrease of its mean values. Secondly, the maximum of the ankle power generation increased slightly, though remaining below average data. Such improvement during terminal stance could be explained like this: firstly, the lengthened muscle delayed the stretch and reflexive response until late stance when push-off occurred; secondly, the muscle could be better mechanically positioned as regards the ankle, in order to generate energy. As presented in Table 2, in the preoperation phase, the ankle was in a position of −44.9° on right side and of −48.3° on left side of dorsiflexion at onset of push-off (Maximum in Stance index), while in the POST1 session it was in a position of 12.2° and 13.1, respectively, remaining close to these values in the second follow-up. This increase in dorsiflexion allows the triceps surae to contract better [23].

However, it is evident from these data that the maximum values of ankle moment (Max ADPM index) and of the generated power at push-off are still low, if compared to the average data. Thus, even if a decrement of the maximum of ankle moment occurred, the main result was the normalization of its timing, the change in the shape of the ankle moment graph after surgery may be related to a better support following lengthening of the gastrocnemius [31]. Then, the slight increment of the generated ankle power demonstrated that gastrocnemius fascia lengthening did not increase functional muscle weakness, a point that is of widespread clinical concern. After 10 months the child was able to keep a better biomechanical position and to walk, even if he revealed residual functional weakness, displayed by limited ankle moment and power. During gait, in fact, force output by plantarflexors is likely much lower than the maximum possible force [20]. Thus, as elicited from the data it is advisable to reinforce muscles more and more by a physiotherapic program, in order to strengthen them. 

In conclusion, this case study highlighted that GA was effective not only to quantify the results of the treatment but also to help preoperative decision making in dealing with CP conditions. The surgical choice was based, in fact, on kinetic ankle data and, in particular, on ankle moment. Without ankle moment data, displayed in PRE session a maximum of ankle moment close to normality in terms of magnitude and so indicative of a satisfactory force of the triceps surae, the surgeon would not have operated the patient, in order to avoid increasing the child's muscle weakness, causing his walking inability. This kinetic information is supplied only by GA and not by clinical evaluation or video analysis. This concept underlines the importance of GA in surgical decision making. 

The analysis demonstrated the positive results of the modified version of Vulpius lengthening technique on all lower limb joints, including the hip, which had never been investigated in the previous studies, and on kinematic and kinetic data obtained by GA at the explored follow-ups (two and ten months). In particular the most important achievement from the treatment described was the child's increased function and independence in community. The main surgeon's concern was about the further weakening of a muscle which was not strong before surgery and which might make the patient unable to walk autonomously. The appearance of crouch gait in the last follow-up may be connected to the increasing hamstring contracture, which generally represents the major factor influencing recurrence of equinus and to the weakening of the soleus, a potential worsening already described in literature.

## Figures and Tables

**Table 1:** Gait parameters and descriptors.

Gait Parameter	Description
*Spatio-temporal parameters*	

Velocity (m/s)	mean velocity of progression, normalised to subject's height
% stance (%gait cycle)	% of gait cycle that begins with initial contact and ends at toe-off of the same limb
Step length	longitudinal distance from one foot strike to the next one, normalised to subject's height
Step width (mm)	medio-lateral distance between the two feet during double support

*Kinematics (degrees)*	

*Pelvic Tilt*	
Mean Pelvic Tilt	mean value of motion at pelvic joint on the sagittal graph (Pelvic Tilt graph) during the gait cycle
ROM Pelvic Tilt	the range of motion at pelvic joint on the sagittal plane (Pelvic Tilt graph) during the gait cycle, calculated as the difference between the maximum and minimum values of the plot
ROM Pelvic Obliquity	the range of motion at pelvic joint on the frontal plane (Pelvic Obliquity graph) during the gait cycle, calculated as the difference between the maximum and minimum values of the plot
ROM Pelvic Rotation	the range of motion at pelvic joint on the transversal plane (Pelvic Rotation graph) during the gait cycle, calculated as the difference between the maximum and minimum values of the plot
*Hip Flex-Extension*	
IC	value of Hip Flexion-Extension angle (hip position on sagittal plane) at initial contact, representing the position of hip joint at the beginning of gait cycle
Min in St	minimum of hip flexion (hip position on sagittal plane) in stance phase, representing the extension ability of hip during this phase of gait cycle
Mean Hip Rotation	mean value of Hip Rotation angle (hip position on transversal plane) during all the gait cycle
*Knee Flex-Extension*	
IC	value of Knee Flexion-Extension angle (knee position on sagittal plane) at initial contact, representing the position of knee joint at the beginning of gait cycle
Min in St	minimum of knee flexion (knee position on sagittal plane) in midstance, representing the extension ability of knee during this phase of gait cycle
Max in Sw	maximum of knee flexion (knee position on sagittal plane) in swing phase, representing the flexion ability of knee joint during this phase of gait cycle
*Ankle Dorsi-Plantarflexion*	
IC	value of the ankle joint angle (on sagittal plane) at the initial contact, representing the position of knee joint at the beginning of gait cycle
Max in St	maximum of ankle dorsiflexion (on sagittal plane) during stance phase, representing the dorsiflexion ability of ankle joint during this phase of gait cycle
ROM in St	the range of motion at ankle joint on the sagittal plane (Ankle Dorsi-plantarflexion graph) during stance phase, calculated as the difference between the maximum and minimum values of the plot during stance phase
Max in Sw	maximum of ankle dorsiflexion (on sagittal plane) during swing phase, representing the dorsiflexion ability of ankle joint in this phase of gait cycle
*Foot progression*	
Mean Foot Progression	mean value of Foot Progression (foot position on the transversal plane) during all the gait cycle

*Kinetics*	

*Ankle Dorsi-plantarflexion Momen* *t (N∗m/Kg)*	
Max ADPM in St	the maximum value of plantarflexion ankle moment during terminal stance (maximum value of positive ankle moment during terminal stance)
*Ankle Power (W/Kg)*	
Max push-off	the maximum value of generated ankle power during terminal stance (maximum value of positive ankle power during terminal stance) representing the push-off ability of the foot during walking
*Knee Flex-Extension Moment (N∗m/Kg)*	
Max KFEM in St	the maximum value of flexor knee moment during terminal stance (maximum value of positive knee moment during terminal stance)
*Knee Power (W/Kg)*	
min in St	the minimum value of absorbed knee power during terminal stance (maximum value of negative knee power during terminal stance)
*Hip Flex-Extension Moment (N∗m/Kg)*	
Max HFEM in St	the maximum value of flexor hip moment during stance (maximum value of positive hip moment during stance)
*Hip Power (W/Kg)*	
Max in St	the maximum value of generated hip power during stance (maximum value of positive hip power during stance)

**Table 2:** Summary of kinematic parameters (mean and standard deviation) for analysed patient (CP patient; right and left side) in the examined sessions and normative data (Control Group, CG).

	*CP patient PRE*		*CP patient POST1*		*CP patient POST2*		CG
			*(2 months)*		*(10 months)*		
	right	left	right	left	right	left	
*Pelvic Tilt (degrees)*							
Mean Tilt	3.8 (0.7)	3.2 (1.9)	11.4 (0.3)*	12.1 (0.7)*	7.9 (0.1)^+^	8.4 (1.5)^+^	8.8 (4.3)
ROM	4.5 (2.7)	5.5 (2.4)	3.3 (0.9)	3.5 (1.1)	3.9 (1.3)	5.5 (0.8)	1.6 (3.6)
*Pelvic Obliquity (degrees)*							
ROM	4.6 (1.1)	5.2 (1.7)	4.1 (0.1)	4.8 (0.1)	4.6 (1.5)	5.2 (0.6)	6.5 (6.9)
*Pelvic Rotation (degrees)*							
ROM	5.4 (0.2)	6.0 (0.3)	4.9 (0.1)	5.6 (0.3)	3.6 (1.4)	4.9 (2.2)	10.7 (5.3)
*Hip Flex-Extension (degrees)*							
IC	38.7 (3.2)	25.9 (1.1)	29.7 (0.1)*	27.2 (1.7)	30.6 (0.5)^+^	28.8 (2.1)	29.5 (4.4)
Min in St	13.5 (2.7)	4.0 (0.5)	9.8 (0.6)	10.9 (0.5)*	5.4 (1.02)^+^	8.3 (1.1)^+^	−8.7 (6.4)
*Hip Ab-Adduction (degrees)*							
ROM	6.1 (0.1)	6.3 (2.9)	3.9 (0.1)	4.7 (0.5)	5.8 (1.9)	6.1 (0.4)	10.7 (3.1)
*Hip Intra-extrarotation (degrees)*							
Mean hip rotation	7.3 (1.7)	8.2 (1.6)	2.6 (0.1)	4.7 (1.3)	5.2 (3.6)	6.7 (0.5)	−1.9 (3.9)
*Knee Flex-Extension (degrees)*							
IC	36.8 (0.8)	26.6 (1.9)	10.2 (0.9)*	8.2 (2.3)*	18.9 (1.5)*^,+^	20.4 (2.8)*^,+^	6.7 (5.5)
Min in St	36.2 (0.5)	20.9 (0.6)	8.7 (3.1)*	7.3 (0.9)*	18.4 (2.2)*^,+^	21.5 (0.5)*^,+^	4.2 (2.1)
Max in Sw	42.3 (2.2)	29.6 (0.8)	37.1 (0.2)	32.0 (1.3)	48.6 (1.1)*^,+^	50.1 (2.5)*^,+^	60.5 (4.7)
*Ankle Dorsi-Plantarflexion (degrees)*							
IC	−48.1 (0.9)	−50.8 (1.8)	3.1 (0.7)*	7.1 (1.4)*	1.9 (0.5)*	4.5 (1.4)*	-0.5 (4.8)
Max in St	−44.9 (1.3)	−48.3 (2.5)	12.2 (0.3)*	13.1(0.4)*	10.0 (2.1)*	15.5 (0.6)*	12.2 (5.5)
Timing of Max in St (%gc)	24 (2)	40 (3)	40 (1)*	58 (2)*	30 (3)*^,+^	32 (2)*^,+^	35 (3)
ROM in St	9.4 (3.5)	4.1 (3.1)	7.4 (2.3)	10.8 (1.9)*	17.5 (3.1)*^,+^	15.4 (0.8)*^,+^	23.4 (4.8)
Max in Sw	−47.6 (0.2)	−47.1 (1.5)	9.1 (2.5)*	13.3 (0.5)*	4.7 (1.3)*^,+^	8.4 (2.7)*^,+^	5.8 (6.5)
*Foot Progression (degrees)*							
Mean	−17.5 (0.2)	−20.1 (3.5)	−15.5 (3.6)	−17.1 (3.3)	−12.4 (1.5)	−14.8 (0.4)	−12.5 (5.8)

Abbreviations: ROM: Range Of Motion; Min: Minimum value; Max: Maximum value; IC: Initial Contact; St: Stance phase; Sw: Swing phase

Sagittal Plane: positive values: Flexion/Dorsiflexion; negative values: Extension/Plantarflexion. Frontal Plane: positive values: up/adduction; negative values: down/abduction. Transversal plane: positive values: intra-rotation; negative values: extrarotation.

*P < .05, PRE versus POST1 and POST2; ^+^ *P* < .05 POST1 versus POST2.

**Table 3:** Summary of kinetic parameters (mean and standard deviation) for analysed patient (CP patient; right and left side) in the examined sessions and normative data (Control Group, CG).

	*CP patient PRE*		*CP patient POST1*		*CP patient POST2*		CG
			*(2 months)*		*(10 months)*		
	right	left	right	left	right	left	
*Ankle Dorsi-Plantarflexion Moment (N∗m/Kg)*							
Max ADPM in St	1.1 (0.1)	0.9 (0.1)	0.6 (0.2)*	0.4 (0.1)*	0.5 (0.2)*	0.6 (0.3)*	1.3 (0.2)
*Ankle Power (W/Kg)*							
Max push-off	0.1 (0.1)	0.1 (0.2)	0.5 (0.2)*	0.3 (0.3)*	0.5 (0.3)*	0.6 (0.1)*	3.9 (1.2)
*Knee Flex-Extension Moment (N∗m/Kg)*							
Max KFEM in St	1.5 (0.1)	0.7 (0.2)	0.3 (0.1)*	0.6 (0.2)*	1.0 (0.1)*	0.5 (0.1)	0.1 (0.2)
*Knee Power (W/Kg)*							
Min in St	−0.8 (0.1)	−0.7 (0.2)	−0.2 (0.1)*	−0.5 (0.2)*	−1.3 (0.2)^+^	−1.0 (0.2)^+^	−0.1 (0.2)
*Hip Flex-Extension Moment (N∗m/Kg)*							
Max HFEM in St	2.1 (0.3)	1.1 (0.2)	1.2 (0.2)*	0.8 (0.4)	0.9 (0.2)*	0.7 (0.1)*	0.9 (0.3)
*Hip Power (W/Kg)*							
Max in St	1.9 (0.2)	0.8 (0.1)	0.6 (0.1)*	0.5 (0.2)	0.7 (0.1)*	0.6 (0.1)	0.8 (0.3)

Abbreviations: Max: Maximum value; St: Stance phase; ADPM: Ankle Dorsi-Plantarflexion moment; KFEM: Knee Flex-Extension

Moment; HFEM: Hip Flex-Extension Moment.

*P < .05, PRE versus POST1 and POST2; ^+^ *P* < .05 POST1 versus POST2.

**Table 4:** Ankle rockers.

*Ankle Rockers description*	
First rocker	It is referred to as the heel rocker. It begins at initial contact and extends through loading response. In normal gait the fulcrum of this rocker is the heel. Posterior protrusion of the heel creates a level equal to 25% of the foot total length and because the ground reaction force acting on this level is passing through the heel at initial contact, its immediate effect is to thrust the entire foot towards the floor. This external moment is resisted by the internal moment of the pretibial muscles. The purpose of the first rocker is shock absorption, that is, to decelerate the body's inertia at initial contact.
Second rocker	In this period the fulcrum has moved from the heel to the centre of the ankle joint as the tibia hinges forward on the stationary foot. The purpose of the second rocker is to control the position of ground reaction force referable to the joints above.
Third rocker	In this period the fulcrum has moved forward from the ankle to the metatarsal heads. The deceleration of the first two rockers is balanced by the acceleration produced by the third one.
